# Unveiling the Rare: A Case of Renal Cell Carcinoma Acrometastasis

**DOI:** 10.7759/cureus.73612

**Published:** 2024-11-13

**Authors:** Carina E Ferraris, Momoko Ishizuka, Gary Schwartz

**Affiliations:** 1 Medicine, Nova Southeastern University Dr. Kiran C. Patel College of Allopathic Medicine, Fort Lauderdale, USA; 2 Orthopedic Surgery, Nova Southeastern University Dr. Kiran C. Patel College of Allopathic Medicine, Fort Lauderdale, USA

**Keywords:** acrometastasis, renal cell carcinoma (rcc), renal cell metastasis, skeletal metastasis, surgical case report

## Abstract

Acrometastasis refers to a rare form of skeletal metastasis affecting sites distal to the knees and elbows, often leading to significant functional impairment and reduced quality of life. This case report details a 65-year-old man with a history of renal cell carcinoma (RCC) who presented with persistent pain and swelling in the distal aspect of his left long finger. An incisional biopsy confirmed the lesion as metastatic RCC. X-ray and MRI assessed the lesion’s progression, guiding the treatment plan. The patient subsequently underwent disarticulation of the distal interphalangeal joint and was followed up one-month post-surgery before relocating and being admitted to hospice care. This report underscores the importance of recognizing acrometastasis as a potential indicator of recurrence in patients with a malignancy history. Although the prognosis for acrometastasis is generally poor, early identification and intervention are essential for improving patient outcomes.

## Introduction

Renal cell carcinoma (RCC) is the most common type of kidney cancer in adults, accounting for 80-85% of all primary renal neoplasms. Approximately 63,000 new cases are diagnosed annually in the United States, with around 14,000 deaths attributed to RCC. RCC primarily affects men aged 50-70, with additional risk factors including obesity, hypertension, chronic renal failure, polycystic kidney disease, African American race, and dialysis [1,2]. Certain hereditary conditions, such as tuberous sclerosis, Von Hippel-Lindau (VHL) disease, and hereditary papillary renal carcinoma, also increase RCC risk [3]. Occupational exposure to substances like asbestos and trichloroethylene has been associated with RCC development. RCC can be classified into hereditary (linked to VHL, tuberous sclerosis complex, or mesenchymal-epithelial transition genes) and nonhereditary forms. Structural changes in both forms occur on the short arm of chromosome 3, with the clear cell carcinoma subtype commonly associated with a 3p deletion [1].

RCC originates in the proximal renal tubular epithelium within the renal cortex. Patients are often asymptomatic in the early stages, especially when the mass is smaller than 3 cm. In about 25% of cases, solid renal masses are incidental findings during routine radiological studies. Symptoms like fever, fatigue, weight loss, back pain, anemia, and hypercalcemia may emerge as the mass grows. Although the classic triad of flank pain, hematuria, and flank mass is widely recognized, it appears in only 10% of patients, typically indicating advanced disease [1].

Skeletal metastasis occurs in up to 30% of advanced-stage RCC cases, often resulting in severe bone pain and reduced quality of life. However, acrometastasis - metastasis to sites distal to the knees and elbows - is rare, representing only about 0.1-0.3% of metastatic osseous lesions [4]. Increased regional blood flow and red marrow density are factors contributing to bone metastasis [1]. The vascularity and red marrow in adult hand bones may explain the relatively higher incidence of hand acrometastasis in this select population [5].

This report presents a case of a patient with a history of RCC who, one-year post-diagnosis, presented with acrometastasis in the left hand, signaling disease recurrence.

## Case presentation

A 65-year-old man with a one-year history of RCC presented with persistent pain and swelling in the distal aspect of the left long finger for the past six months. His baseline functional status was limited due to recurrent pleural effusions and pulmonary issues.

**Figure 1:**
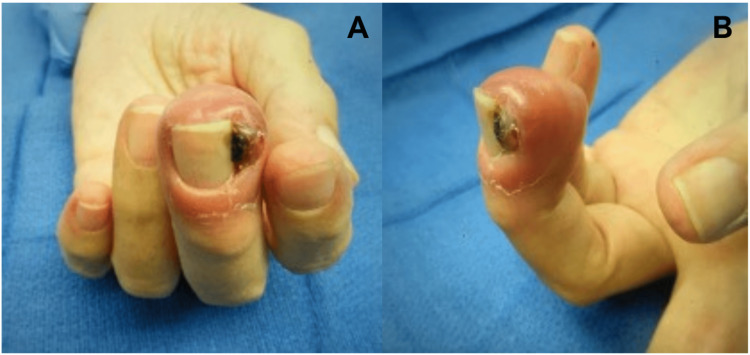
Preoperative appearance Preoperative photographs showing (A) dorsal and (B) lateral views of the left long finger, demonstrating swelling, deformity, and erythema localized to the distal phalangeal region

On examination, tenderness, swelling, and erythema were noted in the distal phalangeal region of the left long finger (Figure 1). There was no tenderness in the shoulder, elbow, or forearm. An incisional biopsy of the lesion in the distal phalanx of the left long finger confirmed metastatic RCC.

**Figure 2:**
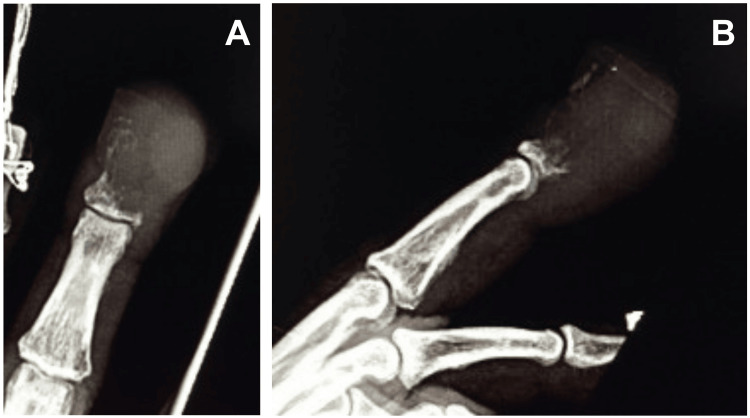
Preoperative radiographs Preoperative radiographs showing (A) anterior-posterior and (B) lateral views of the long finger, illustrating a destructive lesion with mass effect involving the distal phalanx.

X-rays of the left long finger revealed a destructive process with swelling and/or a soft tissue mass involving the distal phalanx. No definite involvement of the middle or proximal phalanx was observed on imaging (Figure 2).

**Figure 3:**
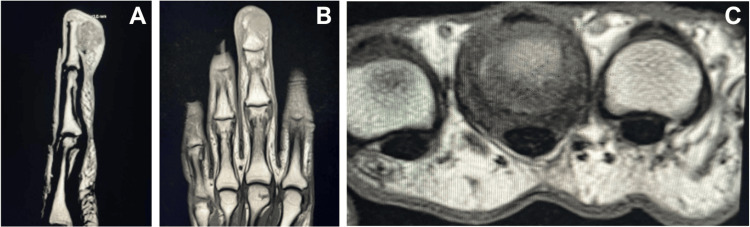
Preoperative MRI Preoperative MRI of the left long finger showing the enhancing subcutaneous mass along the volar aspect with erosive changes, displayed in the (A) sagittal, (B) coronal, and (C) axial planes.

A contrast-enhanced MRI study of the left long finger revealed an enhancing subcutaneous mass measuring 41 × 16 × 12 mm (CC × TRV × AP) along the volar aspect. The lesion appeared mildly hypointense on T1-weighted images and hyperintense on T2-weighted images. It abutted the volar aspect of the distal phalanx, showing mild erosive changes. Additionally, a mild hyperintense marrow signal was observed in the distal phalanx without corresponding T1 hypointensity. No involvement of the middle or proximal phalanx, nor significant joint effusion, was noted (Figure 3).

**Figure 4:**
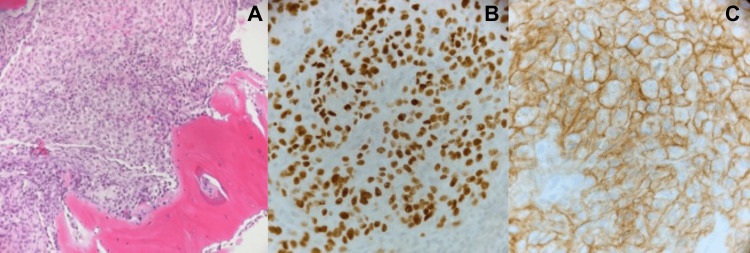
Histopathologic findings (A) H&E staining of the long finger mass, showing tumor invasion into bone. (B) PAX-8 immunostaining and (C) CA IX immunostaining of the mass reveal features consistent with RCC. RCC, renal cell carcinoma

Following an orthopedic hand surgery consultation, the decision was made to perform a distal interphalangeal joint disarticulation of the left long finger. Under general anesthesia with tourniquet control, a fish-mouth incision was made on the dorsal and volar aspects of the left long finger at the level of the distal interphalangeal joint. Dissection revealed the skin, subcutaneous tissue, and extensor mechanism. The radial and ulnar digital nerves, as well as blood vessels, were identified and cauterized. The distal interphalangeal joint was disarticulated and sent for pathology. The pathology report confirmed metastatic RCC (Figure 4). PAX-8 immunostain, a transcription factor, was used to identify thyroid, renal, and gynecologic tumors. The CA IX immunostain, a transmembrane protein, was useful in identifying kidney tumors, particularly clear cell renal carcinoma.

The patient was followed up one month after surgery. During this follow-up, he relocated out of state and was subsequently admitted to a hospice unit.

## Discussion

Pathogenesis

Evidence suggests that acrometastasis occurs through hematogenous rather than lymphatic spread from the primary tumor [6,7]. This may explain why 47% of metastatic hand lesions are associated with primary lung tumors, as the lungs have direct access to systemic circulation [6]. By contrast, other tumor emboli must traverse the hepatic and pulmonary circulations before reaching the brachial artery.

For cancer cells to survive in systemic circulation and initiate metastasis, they must invade sinusoidal vessels and adapt to the bone marrow environment. Here, they establish their own blood supply and promote osteoclastic activity to migrate to the bone surface [8]. Research has identified increased heparanase activity in clear cell RCC as correlated with greater metastasis and angiogenesis [9]. Heparanase, an enzyme that cleaves heparan sulfate proteoglycans in the extracellular matrix and basement membrane, facilitates tumor invasion and metastasis [10]. It also promotes tumor-driven angiogenesis and inflammation [11].

Additionally, the receptor activator of nuclear factor κB ligand (RANKL) plays a significant role in acrometastatic RCC. The RANKL pathway, including its receptor RANK and decoy receptor osteoprotegerin, is well studied in bone remodeling [12]. RANKL has also been shown to trigger migration in RANK-expressing cancer cells [13], with both RANK and RANKL expressed in primary and metastatic RCCs [14]. This pathway may facilitate acrometastasis by enhancing cancer cell migration and osteoclastogenesis.

Diagnosis

The clinical presentation of acrometastasis often includes erythema or other discoloration, pain, swelling, tenderness, warmth, and functional impairment. In more advanced cases, lesions may become ulcerated and bleed [15]. These symptoms can overlap with various differential diagnoses, such as osteomyelitis, gout, infection, and rheumatoid arthritis. Misidentifying acrometastasis as one of these more common pathologies could delay diagnosis and lead to inappropriate treatment. Bony metastases may therefore be challenging to recognize unless clinicians specifically investigate their possibility, highlighting the need for additional diagnostic approaches.

Given the extremely poor prognosis for acrometastasis, prompt diagnosis and treatment are crucial. A collaborative, multidisciplinary approach is essential for managing these patients. Imaging studies of the skeleton should be prioritized for individuals with a known malignancy who report bone pain, particularly those at high risk for metastatic disease. X-rays, CT scans, MRIs, and bone scans should be employed as part of a thorough workup, and biopsies of affected tissues should be obtained when feasible. Radiographic findings showing an osteolytic lesion without periosteal involvement, combined with a history of malignancy, should raise suspicion for acrometastasis [16].

Outcomes/treatment

The presence of acrometastasis is a poor prognostic indicator, with survival rates ranging from 1 to 54 months and mean survival times reported between 12.3 and 14.8 months [17]. This prognosis is heavily influenced by the behavior of the primary tumor, so developing an appropriate treatment plan requires identification of the primary tumor, complete staging, and assessment of the patient’s performance status [6]. Given the rarity of acrometastasis, no standardized treatment protocol exists; management is primarily palliative. The main treatment objectives are pain relief, tumor resection, preservation of function, and enhancement of quality of life [18].

Resection methods must be tailored to each case. Due to the limited soft tissue in the hand, ray resection or amputation is frequently required [6,18]. In some instances, lesions may be too large for resection without causing significant disfigurement and functional impairment. For these cases, options like radiation, curettage, or marginal excision may be considered to provide symptom relief with minimal cosmetic impact [19].

Patients with solitary osseous lesions from RCC, treated with wide resection and nephrectomy, have shown an excellent prognosis. In a study by Jung et al., all eight patients with solitary osseous RCC lesions treated in this manner had a 100% disease-specific survival rate [20]. While it is not clear whether this approach is equally effective for RCC patients with acrometastasis, further research is needed to explore its potential applicability.

## Conclusions

Acrometastasis, particularly from RCC, is a rare occurrence, and routine surveillance is generally not recommended. However, in patients with a history of prior malignancy treatment and persistent, atypical symptoms in the hands or feet, radiological evaluation is crucial to rule out disease recurrence. If recurrence is confirmed, management should involve a multidisciplinary team and be tailored to the stage of recurrence. Treatment options include surgical excision, amputation, radiotherapy, or conservative palliative measures. While acrometastasis typically indicates a poor prognosis, early diagnosis and intervention may improve long-term survival and symptom management.
